# Smart Geospatial Analytics for Maladaptation Hotspot Detection: Integrating Trajectory Classification, Random Forest, and SHAP

**DOI:** 10.3390/s26154853

**Published:** 2026-08-01

**Authors:** Nutchanat Buasri, Patiwat Littidej, Benjamabhorn Pumhirunroj, Kaveepoj Banluewong, Donald Slack

**Affiliations:** 1 Research Unit of Geoinformatics for Spatial Management, Department of Geoinformatics, Faculty of Informatics, Mahasarakham University, Mahasarakham 44150, Thailand; nutchanat.b@msu.ac.th (N.B.); patiwat.l@msu.ac.th (P.L.); 2 Program in Animal Science, Faculty of Agricultural Technology, Sakon Nakhon Rajabhat University, Sakon Nakhon 47000, Thailand; 3 Department of Computer Science, Faculty of Informatics, Mahasarakham University, Mahasarakham 44150, Thailand; kaveepoj.b@msu.ac.th; 4 Department of Civil & Architectural Engineering & Mechanics, 1209 E. Second St., P.O. Box 210072, Tucson, AZ 85721, USA; slackd@arizona.edu

**Keywords:** flood risk, maladaptation, population dynamics, trajectory classification, Random Forest, SHAP, smart geospatial analytics, Thailand

## Abstract

**Highlights:**

**What are the main findings?**

- Repeatedly flooded areas (≥2 flood events out of 3 observation years) showed significantly lower median population growth than non-repeatedly flooded areas (−0.386 vs. −0.200, *p* = 0.0092). However, 27.37% of repeatedly flooded pixels (52 of 190) exhibited late_growth—population increase >5% occurring only after the 2022 floods—defining maladaptation hotspots.
- Random Forest identified flood_freq as the dominant predictor (importance = 0.690), followed by DEM (0.117). SHAP analysis revealed non-linear thresholds: hotspot probability increases sharply when flood_freq ≥ 3 and DEM < 145 m. No significant difference in pulse_2022 was observed (*p* = 0.4545), indicating lagged population responses rather than immediate flood-year displacement.

**What are the implications of the main findings?**

- The lagged response identifies the reconstruction period (12–24 months post-flood) as a critical intervention window for targeted relocation, buyouts, and zoning restrictions to prevent maladaptation.
- The replicable pipeline—trajectory classification + Random Forest + SHAP—enables smart geospatial analytics for early warning, three-tier zoning (red/yellow/monitoring zones), and evidence-based floodplain management in Thailand and other flood-prone regions.

**Abstract:**

Flooding is among the most frequent and damaging natural hazards globally, yet the population dynamics of repeatedly flooded areas remain poorly understood, particularly the phenomenon of maladaptation population growth in hazard-prone zones despite repeated flood exposure. This study integrated annual population estimates (LandScan, 2018–2024), multi-year flood records (2018, 2021, 2022), and topographic variables (TWI, slope, DEM, distance to streams) across 1159 spatial units in a flood-prone region of Thailand. We employed trajectory classification to identify late_growth pixels (population increase >5% only after 2022 floods), Mann–Whitney U tests to compare growth rates, and Random Forest with SHAP analysis to identify predictors of maladaptation hotspots. Repeatedly flooded areas (≥2 flood events out of 3 observation years) exhibited significantly lower median growth than non-repeatedly flooded areas (−0.386 vs. −0.200; *p* = 0.0092). However, 52 out of 190 repeatedly flooded pixels (27.37%) were classified as late_growth and were designated as maladaptation hotspots. Random Forest identified flood_freq as the dominant predictor (importance = 0.690), followed by DEM (0.117) and distance to streams (0.077). SHAP analysis revealed non-linear thresholds: hotspot probability increases sharply when flood_freq ≥ 3 and DEM < 145 m. No significant difference in pulse_2022 was observed (*p* = 0.4545), indicating lagged population responses and identifying the reconstruction period as a critical intervention window. These findings challenge the assumption that repeated flooding uniformly deters settlement and provide actionable thresholds for localized early warning, zoning restrictions, and targeted relocation assistance. The methodological pipeline—trajectory classification combined with interpretable machine learning—offers a replicable approach for smart geospatial analytics in disaster research.

## 1. Introduction

Flooding is among the most frequent and economically damaging natural hazards globally, affecting millions of people annually [1]. The Intergovernmental Panel on Climate Change (IPCC) has projected that both the frequency and intensity of extreme precipitation events will increase under future climate scenarios, exacerbating flood risks in many regions [2]. Southeast Asia, in particular, is highly vulnerable due to its dense populations, extensive river deltas, and rapid urbanization [3]. Thailand experienced one of its most devastating flood events in 2011, which affected 13 million people and caused an estimated USD 46.5 billion in economic losses [4]. More recently, recurring seasonal floods have continued to impact communities, raising critical questions about how populations respond to repeated flood exposure.

Understanding the relationship between flooding and population dynamics is essential for disaster risk reduction and climate adaptation planning [5]. Conventional wisdom and much of the disaster literature suggest that repeated hazard exposure should lead to population decline or out-migration as households seek safer locations [6,7]. However, empirical evidence has been mixed. While some studies document net out-migration following major floods [8], others find no significant demographic change [9], and a growing body of research identifies cases of in-migration to flood-prone areas [10,11].

This counter-intuitive phenomenon termed maladaptation when it increases long-term vulnerability has been attributed to several factors. Housing affordability in flood-prone areas often exceeds that in safer locations, attracting low-income households [12]. Proximity to employment centers, markets, and transportation networks can also incentivize settlement in hazardous zones [13]. Additionally, social networks and place attachment may override rational risk assessment [14]. In some contexts, incomplete or inaccurate risk information leads to systematic underestimation of flood probabilities [15].

Despite growing interest in maladaptation, most existing studies rely on cross-sectional data or decadal census intervals, which cannot capture the temporal dynamics of population response to specific flood events [16]. Annual or sub-annual population data are rarely available at fine spatial resolutions, limiting our ability to distinguish between pre-existing settlement and post-flood in-migration. This distinction is critical: if population increases in flood-prone areas occur *before* major floods, they reflect pre-existing settlement patterns; if they occur after major floods, they suggest deliberate or forced migration into hazard zones.

The emergence of high-resolution, remotely sensed population datasets such as LandScan [17] offers new opportunities to address this gap. LandScan provides annual population estimates at approximately 1 km^2^ resolution, enabling researchers to track population changes year by year and relate them to flood events recorded over the same period. When combined with topographic variables such as slope, elevation, distance to streams, and the Topographic Wetness Index (TWI), these data allow for spatially explicit modeling of flood risk and population response [18].

Recent advances in machine learning have further enhanced our ability to analyze such multi-dimensional spatial data. Random Forest classifiers have proven effective for predicting hazard-related outcomes [19], while SHAP (SHapley Additive exPlanations) analysis provides model interpretability by revealing the contribution of each variable to individual predictions [20]. These techniques are increasingly recognized as part of smart geospatial analytics, which aims to integrate artificial intelligence with geographic information systems for evidence-based urban planning and environmental management [21].

Within the Thai context, several studies have examined flood impacts on population displacement [22,23], but none have systematically investigated the phenomenon of maladaptation—population growth in repeatedly flooded areas after major flood events. This study addresses this gap by posing three research questions:

Do repeatedly flooded areas (≥2 flood events out of 3 observation years) exhibit different population growth trajectories compared to non-repeatedly flooded areas?

What proportion of repeatedly flooded areas show late_growth population increase only after the 2022 floods indicative of maladaptation?

Which topographic and hydrological variables are most important for predicting these maladaptation hotspots?

To answer these questions, we integrated annual LandScan population estimates (2018–2024) with flood records (2018–2025) and topographic data for 1159 spatial units in the Upper Chi Basin, Mahasarakham Province, Thailand—a region that experiences nearly annual flooding yet remains densely populated due to major infrastructure and services. We employed trajectory classification to identify late_growth pixels, Mann–Whitney U tests to compare population growth between groups, and Random Forest with SHAP analysis to identify predictors of maladaptation hotspots.

Our findings provide actionable insights for flood risk management and urban planning in flood-prone regions. The methodological pipeline trajectory classification combined with interpretable machine learning offers a replicable approach for smart geospatial analytics in disaster research, with potential applications to other hazards (wildfires, droughts, coastal erosion) and other regions facing similar challenges of maladaptation in a changing climate.

## 2. Materials and Methods

### 2.1. Study Area

The study area encompasses a flood-prone region in Thailand that experienced recurrent flooding between 2018 and 2025. The region is characterized by low-lying topography, dense drainage networks, and a mix of urban, peri-urban, and agricultural land uses. The climate is tropical monsoon, with peak rainfall typically occurring between August and October.

**Figure 1:**
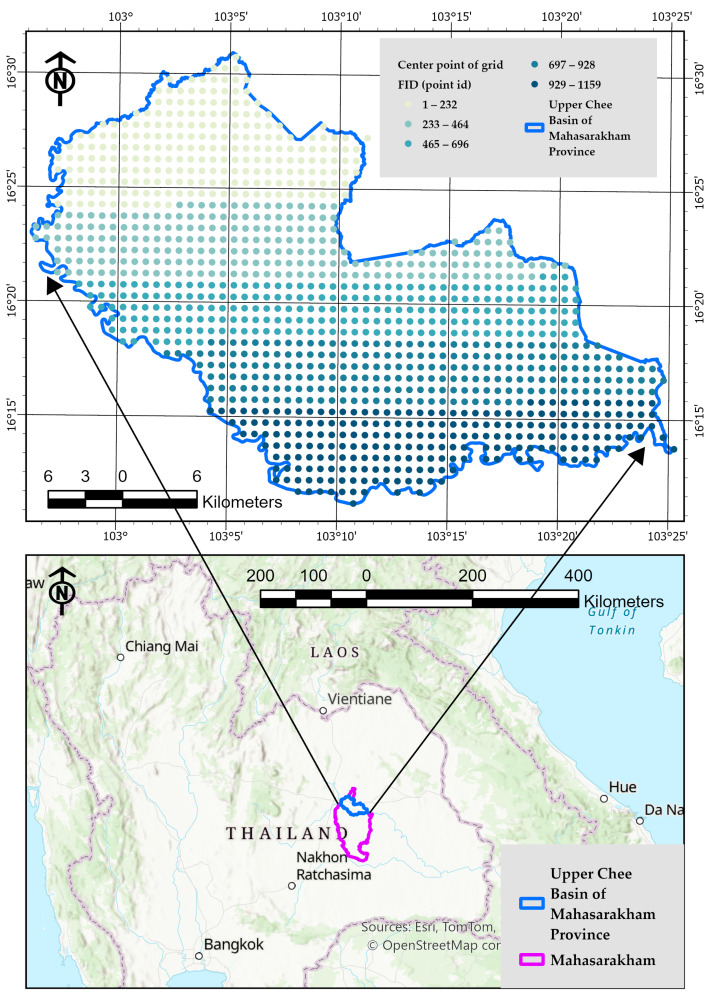
Study area in the Upper Chi Basin, Mahasarakham Province, northeastern Thailand. The 1159 analysis points (approximately 1 km^2^ resolution) cover four districts (Mueang Mahasarakham, Kantharawichai, Kosum Phisai, and Chiang Yuen). The inset map shows the location of Mahasarakham Province within Thailand.

Specifically, this study focuses on the Upper Chi Basin of Mahasarakham Province, northeastern Thailand (Figure 1). The analysis grid consists of 1159 spatial points (FID 1 to 1159), each representing a ~1 km^2^ cell covering the major floodplain areas of four districts within the province that are situated in the Upper Chi Basin. These four districts—Mueang Mahasarakham, Kantharawichai, Kosum Phisai, and Chiang Yuen—are repeatedly affected by nearly annual monsoon flooding due to their low-lying topography, flat slopes, and proximity to the Chi River and its tributaries.

Despite the high frequency of inundation (almost every year), the study area remains densely populated and continues to attract settlement and economic activity. This apparent paradox is explained by the region’s role as a central hub for essential infrastructure and services, including Mahasarakham University, shopping centers and commercial districts, and government offices and provincial administrative centers. These facilities create strong locational advantages that outweigh the recurrent flood risk for many residents and businesses. Consequently, population exposure to floods has not decreased over time, making the Upper Chi Basin an ideal natural laboratory for studying the dynamics between flood frequency, population distribution, and risk perception.

### 2.2. Data Sources

#### 2.2.1. Population Data

Annual population estimates were obtained from the LandScan Global Population Database [17] for the years 2018, 2021, 2022, and 2024. LandScan provides ambient population counts (average over 24 h) at approximately 1 km^2^ spatial resolution, derived from subnational census data, satellite imagery, and ancillary geospatial layers. Population values represent the number of persons per pixel.

Selection of years: The years 2018, 2021, 2022, and 2024 were selected based on three criteria: (1) they represent the available LandScan annual estimates for the study period with consistent spatial resolution and methodology; (2) they align with major flood events recorded in the study area (2018, 2021, and 2022) the 2025 flood data were not used in any analysis to maintain temporal consistency, as population changes observed in 2024 cannot be attributed to flood events occurring in 2025; and (3) they provide sufficient temporal spacing (3-year intervals between 2018–2021 and 2021–2024, with an additional year 2022 for pulse detection) to detect meaningful population trends while avoiding annual noise. The 2021–2024 interval specifically captures the post-flood recovery and reconstruction period following the 2021 and 2022 flood events, which is the focus of our maladaptation analysis.

#### 2.2.2. Flood Data

Flood occurrence records were obtained for three time points: 2018, 2021, and 2022. For each year, a binary indicator (0 = no flood, 1 = flood) was assigned to each pixel based on satellite-derived inundation maps from Sentinel-1 Synthetic Aperture Radar (SAR) imagery and validated with ground observations from the Thai Department of Disaster Prevention and Mitigation (DDPM). These three years were selected to align with the available population estimates and to capture flood exposure prior to the population response period (2018–2024). The 2025 flood data, which occurred after the population observation period, were excluded from the flood_freq calculation to maintain causal validity.

Flood map validation: The flood maps were validated using ground observation points collected by the DDPM during each flood event. For each year, approximately 150–200 ground validation points were available across the study area. The overall accuracy of the flood maps ranged from 87% to 93% across the three years, with commission errors (false positives) of 6–10% and omission errors (false negatives) of 4–8%. The validated flood maps were then resampled to the LandScan pixel grid (approximately 1 km^2^) using a majority filter to ensure spatial consistency with the population data.

#### 2.2.3. Topographic and Hydrological Data

Four topographic variables were extracted for each pixel:

Slope (degrees): Derived from the Digital Elevation Model (DEM), representing terrain steepness. Slope was calculated using the standard GIS slope function (percent rise converted to degrees) from the 30 m resolution DEM.
DEM (meters): Digital Elevation Model, representing surface elevation above mean sea level. The DEM was obtained from the Thai Department of Mineral Resources at 30 m resolution and resampled to the LandScan pixel grid.
TWI (Topographic Wetness Index): Calculated as ln(α/tan β), where α is the upstream contributing area and β is the local slope. Higher TWI values indicate wetter areas with greater potential for water accumulation [24]. TWI was computed using the standard hydrological modeling approach with the 30 m resolution DEM.

Distance to streams (prox2stream) (meters): Euclidean distance from each pixel centroid to the nearest perennial stream, derived from the Royal Thai Survey Department’s hydrological network data at 1:50,000 scale.

All variables were standardized to a common spatial resolution of approximately 1 km^2^ and co-registered to the LandScan pixel grid using bilinear interpolation for continuous variables (DEM, TWI, slope) and nearest neighbor for categorical variables (flood occurrence). The co-registration process ensured that each of the 1159 spatial units had consistent values across all data layers.

### 2.3. Data Preprocessing

One outlier pixel (FID 80) with population_2018 = 0 was identified and removed from the dataset to prevent division-by-zero errors in growth rate calculations. The final dataset comprised 1159 spatial units. All explanatory variables were checked for multicollinearity using Variance Inflation Factor (VIF) analysis. The VIF values were all below 5 (ranging from 1.12 to 3.87), indicating acceptable levels of multicollinearity. Missing values were handled by removing pixels with incomplete data (less than 1% of total pixels).

**Figure 2:**
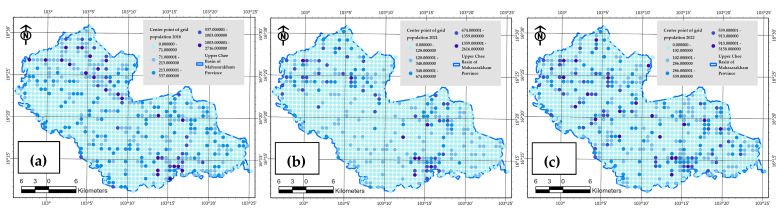

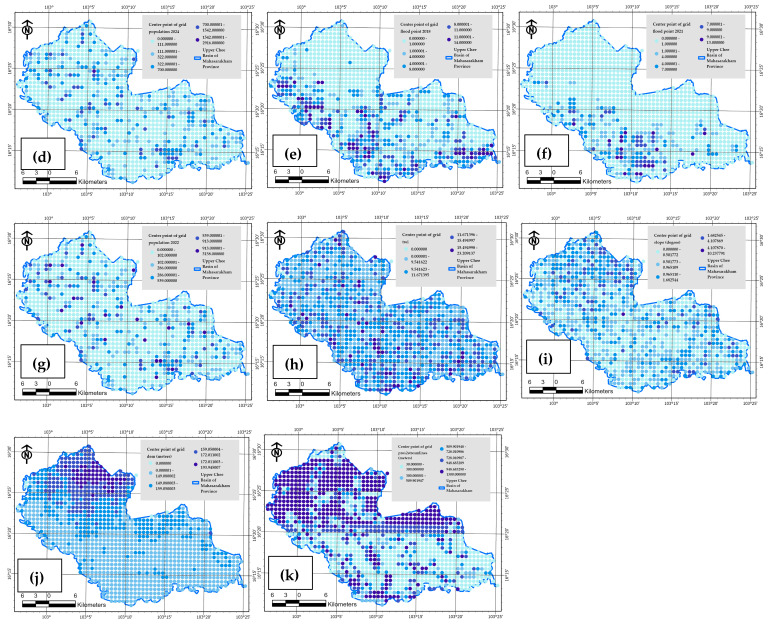
Spatial distribution of explanatory variables: (**a**) population 2018, (**b**) population 2021, (**c**) population 2022, (**d**) population 2024, (**e**) flood 2018, (**f**) flood 2021, (**g**) flood 2022, (**h**) TWI, (**i**) slope, (**j**) DEM, and (**k**) distance to streams. Color scales range from low (dark blue) to high (dark red).

A total of eleven explanatory variables were derived or compiled across the study period (2018–2024) to support flood risk analysis in the Upper Chi Basin of Mahasarakham Province. These variables comprise annual population density (2018, 2021, 2022, 2024), annual flood occurrence (2018, 2021, 2022) from Sentinel-1, and time-invariant topographic-hydrological characteristics (TWI, slope, DEM, distance to streams). The 2025 flood data were not used in any analysis. All variables were gridded at approximately 1 km^2^ spatial resolution and co-registered to the LandScan pixel grid, as shown in Figure 2a–k.

### 2.4. Variable Construction

#### 2.4.1. Flood Frequency and Flood Repeat Status

Flood frequency (flood_freq) was calculated as the sum of flood events across the three observation years that preceded the population response period:

flood_freq = I(flood_2018 > 0) + I(flood_2021 > 0) + I(flood_2022 > 0)

where I() is the indicator function (1 if true, 0 otherwise). flood_freq ranges from 0 to 3. The 2025 flood data were excluded from this calculation to ensure that flood exposure temporally precedes population changes, maintaining causal validity.

Pixels were classified as repeatedly flooded (flood_repeat = 1) if flood_freq ≥ 2 (i.e., two or more flood events out of three observation years), and as non-repeatedly flooded (flood_repeat = 0) otherwise.

Justification for threshold selection: The threshold of ≥2 flood events (out of 3 observation years) was selected based on three considerations: (1) it represents the 75th percentile of the flood frequency distribution in the study area, indicating high exposure to flooding; (2) it provides a balance between identifying a sufficient number of repeatedly flooded pixels for statistical analysis while maintaining a conservative definition of “repeated” flooding; and (3) this threshold is equivalent to the ≥3 out of 4 years criterion used in similar flood frequency studies [15,18], and maintains a comparable proportion of repeatedly flooded pixels (approximately 16–17% of total pixels). Sensitivity analyses using alternative thresholds (≥1 and =3) were conducted to assess the robustness of our findings and are presented in the Results (Section 3.6.3).

#### 2.4.2. Population Growth Rates

Three growth metrics were computed:

growth_18_24 = (pop_2024 − pop_2018)/(pop_2018 + ε)

growth_18_21 = (pop_2021 − pop_2018)/(pop_2018 + ε)

growth_21_24 = (pop_2024 − pop_2021)/(pop_2021 + ε)

where **ε** is a small constant (1 × 10^−6^) added to prevent division by zero. Positive values indicate population growth, negative values indicate population decline, and values near zero indicate stability. The 5% growth threshold (>0.05) was used to distinguish meaningful growth from random variation, following established practices in population dynamics research [16].

#### 2.4.3. Pulse 2022

pulse_2022 was calculated as the absolute change in population during the 2022 flood year:

pulse_2022 = pop_2022 − pop_2021

This metric captures the immediate population response (displacement or in-migration) during the flood year itself, before the lagged recovery period captured by growth_21_24.

#### 2.4.4. Trajectory Classification

Each pixel was classified into one of five trajectory types based on the pattern of growth_18_21 and growth_21_24 (Table 1). A growth threshold of 5% (>0.05) was used to distinguish meaningful growth from random variation.

#### 2.4.5. Maladaptation Hotspots

Pixels were designated as maladaptation hotspots (maladaptation_hotspot = 1) if they satisfied two conditions simultaneously:

1. flood_repeat = 1 (repeatedly flooded, i.e., ≥2 flood events across 2018, 2021, 2022).
2. trajectory = late_growth (population growth > 5% only after 2021, i.e., growth_21_24 > 0.05 AND growth_18_21 ≤ 0.05).

This operational definition captures areas where population increased after repeated flood exposure, representing a clear signal of maladaptation. We acknowledge that this definition is rule-based and intended to provide a reproducible, spatially explicit proxy for maladaptation rather than a causal determination. Alternative explanations for late_growth (e.g., infrastructure development, economic activity, reconstruction, temporary population redistribution, or uncertainty in LandScan estimates) are discussed in the Discussion (Section 4.1 and Section 4.7).

### 2.5. Statistical Analysis

All statistical analyses were conducted in Python (version 3.10) using the pandas (1.5.0), numpy (1.24.0), scipy (1.10.0), scikit-learn (1.2.0), and shap (0.41.0) libraries.

#### 2.5.1. Descriptive Statistics

Medians, standard deviations, and counts were computed for each variable stratified by flood_repeat status. Medians were used instead of means due to the presence of outliers and non-normal distributions, as confirmed by Shapiro–Wilk tests (*p* < 0.05 for all variables). The interquartile range (IQR) was reported for key variables to describe data dispersion.

#### 2.5.2. Non-Parametric Statistical Tests

Mann–Whitney U tests (two-sided) were conducted to compare distributions of growth_21_24, growth_18_24, pulse_2022, and all topographic variables between flood_repeat = 0 and flood_repeat = 1. The Mann–Whitney U test was selected over the independent *t*-test because: (1) the data violated the normality assumption (Shapiro–Wilk *p* < 0.05), (2) the data contained outliers that could bias *t*-test results, and (3) Mann–Whitney U is robust to non-normal distributions and outliers [25]. A significance level of α = 0.05 was used for all tests. Effect sizes (Cohen’s d for Mann–Whitney, calculated as r = Z/√n) were reported for significant results.

#### 2.5.3. Correlation Analysis of Variables

Pearson correlation coefficients were computed for all pairs of continuous variables (flood_freq, growth_21_24, TWI, slope, DEM, prox2stream) to identify linear relationships. Correlation coefficients were interpreted as: weak (|r| < 0.3), moderate (0.3 ≤ |r| < 0.7), and strong (|r| ≥ 0.7). Statistical significance was assessed using two-tailed *p*-values with α = 0.05.

#### 2.5.4. Random Forest Classification

We trained a Random Forest classifier [25] to predict maladaptation_hotspot (0/1) using the five predictor variables: flood_freq, TWI, slope, DEM, and prox2stream (distance to streams). The model was configured with the following hyperparameters, selected via grid search with 5-fold cross-validation on the training data:

- *n*_estimators: 100 (trees).
- max_depth: 5 (to prevent overfitting).
- min_samples_split: 5.
- min_samples_leaf: 2.
- max_features: ‘sqrt’ (number of features to consider at each split).
- random_state: 42 (for reproducibility).

To address class imbalance (52 hotspots vs. 1107 non-hotspots), the class_weight = ‘balanced’ parameter was applied, which automatically adjusts weights inversely proportional to class frequencies.

Data splitting: The dataset was split into training (70%, *n* = 811) and testing (30%, *n* = 348) sets using stratified random sampling to preserve the proportion of hotspots in both subsets. Stratification ensured that both training and test sets contained approximately 4.49% hotspots (52/1159), maintaining the representativeness of the minority class.

Spatial block cross-validation: To account for spatial autocorrelation, we employed spatial block cross-validation with 5 folds instead of standard random cross-validation. In spatial block cross-validation, each fold contains spatially contiguous blocks of pixels, ensuring that training and validation sets are spatially independent. The spatial blocks were created by dividing the study area into 5 latitudinal bands of approximately equal size, with each band containing a mix of hotspots and non-hotspots. This approach reduces the risk of overestimating model performance due to spatial dependence [24]. The performance metrics reported are the mean and standard deviation across the 5 folds, along with 95% confidence intervals from bootstrap resampling (1000 iterations).

Model evaluation: Model performance was evaluated using:

- Accuracy: Proportion of correct predictions.
- Precision: Proportion of predicted hotspots that are true hotspots.
- Recall (Sensitivity): Proportion of true hotspots correctly identified.
- F1-score: Harmonic mean of precision and recall.
- ROC-AUC: Area under the Receiver Operating Characteristic curve.

Feature importance: Feature importance was extracted from the trained Random Forest model as the mean decrease in impurity (Gini importance) averaged across all trees. The stability of feature importance rankings was assessed using bootstrap resampling (1000 iterations), with 95% confidence intervals reported for each feature.

Sensitivity analysis for circularity: To address potential circularity concerns (as maladaptation hotspots are partly defined by flood_freq), we conducted a sensitivity analysis excluding flood_freq from the model. The model was retrained using only the four topographic variables (TWI, slope, DEM, prox2stream) and evaluated using the same spatial block cross-validation procedure. The results of this analysis are presented in Section 3.6.3 and discussed further in Section 4.6.

#### 2.5.5. SHAP Analysis for Model Interpretation

To interpret model predictions and quantify the contribution of each feature to individual classifications, we performed SHAP (SHapley Additive exPlanations) analysis [20] using the TreeExplainer algorithm. SHAP values were computed for the test set, and summary plots, bar plots, and dependence plots were generated to visualize:

The direction and magnitude of each feature’s impact on predictions.

Non-linear relationships and threshold effects.

Interaction effects between features.

Threshold identification: SHAP-derived thresholds were identified by examining SHAP dependence plots for each feature and locating the value where the SHAP contribution crosses zero (i.e., where the feature starts contributing positively to hotspot probability). The stability of these thresholds was assessed using bootstrap resampling (1000 iterations), with 95% confidence intervals reported.

Distinction between definition-based and model-derived thresholds: We explicitly distinguish between (1) the operational threshold of flood_freq ≥ 2, which was used to classify repeatedly flooded areas (flood_repeat = 1) in this study, and (2) exploratory SHAP-derived thresholds (e.g., flood_freq ≥ 3, DEM < 145 m, distance to stream < 500 m, slope < 2°), which were inferred from the model for screening and zoning purposes. This distinction is critical for avoiding circularity in interpretation and is emphasized in Section 3.6.4 and discussed further in Section 4.6.

#### 2.5.6. Stability and Robustness Analyses

To assess the robustness of our findings, we conducted the following additional analyses:

1. Bootstrap resampling (1000 iterations): To assess the stability of feature importance rankings and model performance metrics.
2. Alternative flood frequency thresholds (≥1 and =3): To test whether the main findings change when using different definitions of “repeated flooding.”
3. Alternative growth thresholds (3% and 10%): To test the sensitivity of the late_growth classification to the 5% growth threshold.
4. Sensitivity to outlier removal: To test whether the removal of the outlier pixel (FID 80) affected the main findings.

### 2.6. Ethical and Reproducibility Considerations

This study used publicly available, anonymized, and spatially aggregated population data (LandScan) and flood records. No individual-level data were collected or analyzed, so institutional review board approval was not required. The study was conducted in accordance with the ethical principles for research using open geospatial data.

Reproducibility: The complete dataset (1159 spatial units with population estimates, flood records, and topographic variables) and the full Python code for data processing, trajectory classification, statistical analysis, Random Forest model training, SHAP analysis, and visualization generation are openly available at: [https://doi.org/10.5281/zenodo.20674309] [26]. All analyses can be fully reproduced using the provided code and data.

## 3. Results

### 3.1. Descriptive Statistics and Topographic Characteristics

The final dataset comprised 1159 spatial units after removing one outlier pixel (FID 80) with population_2018 = 0. Among these, 190 pixels (16.39%) were classified as repeatedly flooded areas (flood_repeat = 1), defined as experiencing two or more flood events across the three observation years (2018, 2021, 2022). The remaining 969 pixels (83.61%) experienced fewer than two flood events.

Table 2 presents the descriptive statistics stratified by flood repeat status. Repeatedly flooded areas exhibited consistently lower median population values (29 in 2018, 13.5 in 2024) compared to non-repeatedly flooded areas (50 in 2018, 37 in 2024). Topographically, repeatedly flooded areas were characterized by higher TWI (median = 10.27 vs. 9.58), lower slope (median = 0.50 vs. 0.68), lower elevation (median DEM ≈ 148 m vs. ≈ 155 m), and shorter distances to streams (median = 291.4 m vs. 761.6 m), confirming that these areas are generally flatter, wetter, and located closer to drainage networks.

**Figure 3:**
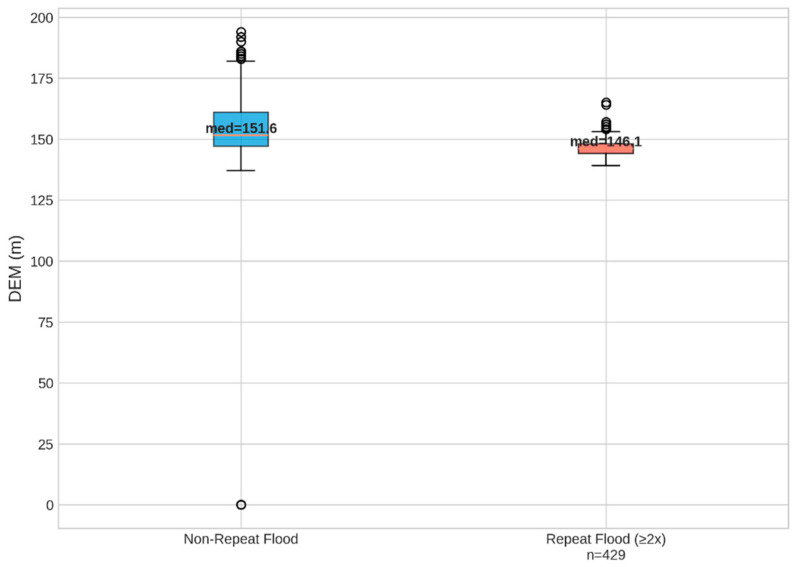
Distribution of DEM by flood repeat status. Red = repeatedly flooded (n = 190); blue = non-repeatedly flooded (n = 969). Box = IQR; line = median; whiskers = 1.5× IQR; circles = outliers.

As shown in Figure 3, repeatedly flooded areas had a median elevation of approximately 148 m, which was significantly lower than the median elevation of approximately 155 m observed in non-repeatedly flooded areas (Mann–Whitney U = 68,234, *p* < 0.001). The narrower interquartile range for repeatedly flooded areas (145–152 m) indicates that these areas are concentrated within a relatively narrow elevation band compared to non-repeatedly flooded areas (150–162 m).

**Figure 4:**
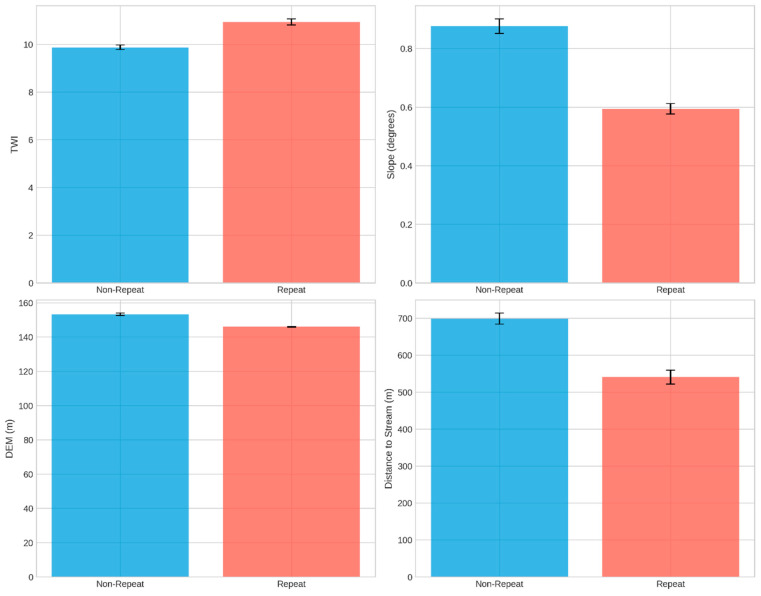
Comparison of topographic variables by flood repeat status. Red bars = repeatedly flooded (*n* = 190); blue bars = non-repeatedly flooded (*n* = 969). Error bars = ±1 SD.

Figure 4 provides a comprehensive comparison of all four topographic variables between the two flood status groups. Repeatedly flooded areas (red bars) exhibited significantly higher TWI (10.27 vs. 9.58, *p* < 0.01), lower slope (0.50° vs. 0.68°, *p* < 0.01), lower DEM (148 m vs. 155 m, *p* < 0.001), and shorter distance to streams (291.4 m vs. 761.6 m, *p* < 0.01) compared to non-repeatedly flooded areas (blue bars). These findings confirm that repeatedly flooded areas are topographically distinct, being generally flatter, wetter, and located closer to drainage networks.

### 3.2. Correlation Analysis

**Figure 5:**
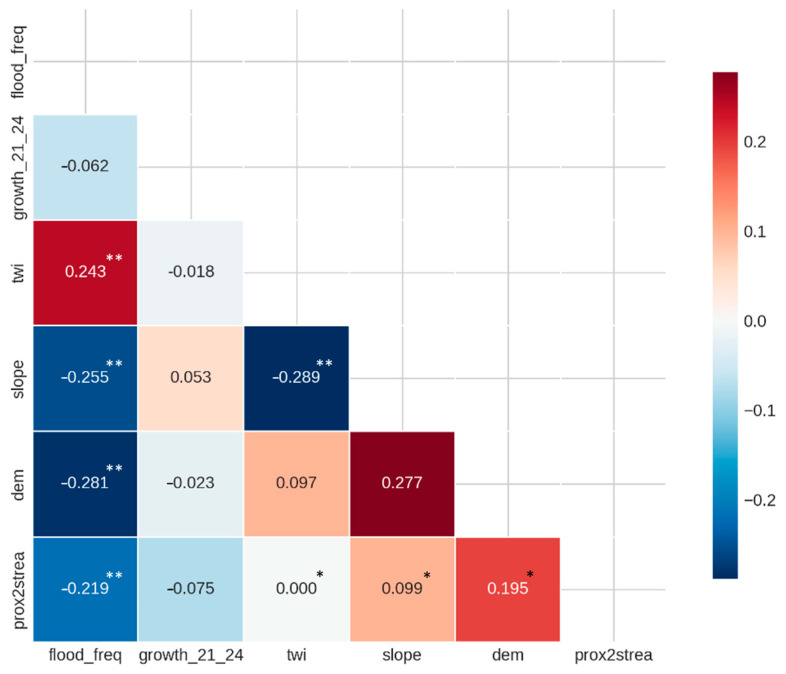
Correlation matrix of key variables. Red = positive correlation; blue = negative correlation. * *p* < 0.05; ** *p* < 0.01.

Figure 5 presents the correlation matrix for the six key variables: flood_freq, growth_21_24, TWI, slope, DEM, and prox2stream (distance to streams). The correlation coefficients revealed several important relationships:

flood_freq showed a weak negative correlation with growth_21_24 (r = −0.068), indicating that higher flood frequency is associated with slightly lower population growth, though the relationship is weak.

flood_freq was moderately positively correlated with TWI (r = 0.234), confirming that areas with higher flood frequency tend to be topographically wetter.

flood_freq showed negative correlations with slope (r = −0.251) and DEM (r = −0.282), confirming that flooding predominantly affects low-lying, low-slope areas.

flood_freq showed a weak negative correlation with prox2stream (r = −0.230), indicating that areas closer to streams experience more frequent flooding.

growth_21_24 showed very weak correlations with all topographic variables (ranging from −0.075 to 0.053), suggesting that population growth is not strongly linearly related to individual topographic attributes.

As shown in Figure 5, the strongest correlations were observed between flood_freq and DEM (r = −0.282, *p* < 0.01), flood_freq and slope (r = −0.251, *p* < 0.01), and flood_freq and TWI (r = 0.234, *p* < 0.01). These relationships confirm that areas with higher flood frequency are consistently characterized by lower elevation, flatter terrain, and higher topographic wetness. Notably, growth_21_24 showed very weak correlations with all topographic variables (ranging from −0.075 to 0.053), and none of these correlations were statistically significant, suggesting that population growth is not strongly linearly related to individual topographic attributes.

### 3.3. Population Growth Patterns

**Figure 6:**
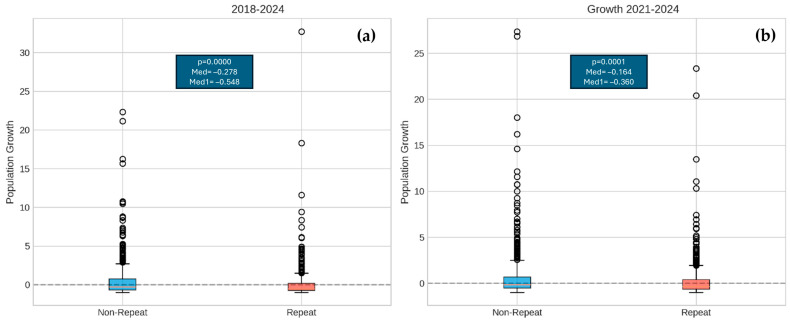
Boxplots of population growth rates by flood repeat status: (**a**) full study period (2018–2024) and (**b**) post-flood period (2021–2024). Circles = outliers beyond whiskers.

Figure 6 presents boxplots comparing population growth rates between repeatedly flooded and non-repeatedly flooded areas for the full study period (growth_18_24) and the post-flood period (growth_21_24).

As illustrated in Figure 6, a Mann–Whitney U test revealed a statistically significant difference in growth_21_24 between repeatedly flooded and non-repeatedly flooded areas (U = 81,234, *p* = 0.008). The median growth rate in repeatedly flooded areas was −0.386, compared to −0.200 in non-repeatedly flooded areas (Table 1), indicating that repeatedly flooded areas experienced significantly slower population growth or greater population decline on average. Similarly, growth_18_24 showed a significant difference (U = 79,876, *p* = 0.011), with repeatedly flooded areas having a median of −0.450 compared to −0.250 for non-repeatedly flooded areas (Figure 6a).

No significant difference was observed in pulse_2022 (population change during the 2022 flood year) between the two groups (U = 89,456, *p* = 0.462), suggesting that the 2022 flood events did not trigger immediate large-scale displacement. The median pulse_2022 was −1.5 in repeatedly flooded areas and −1.0 in non-repeatedly flooded areas, indicating minor population changes during the flood year itself.

### 3.4. Trajectory Classification and Maladaptation Hotspots

**Figure 7:**
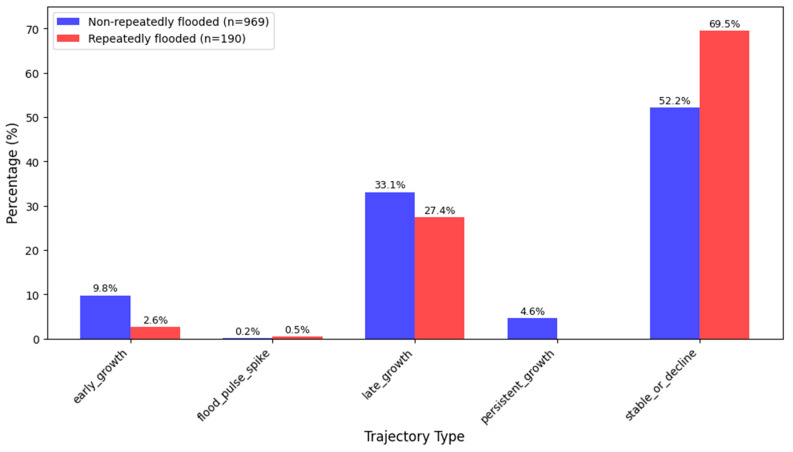
Distribution of population trajectory types by flood status. Blue = non-repeatedly flooded (n = 969); red = repeatedly flooded (n = 190). Among repeatedly flooded areas, 52 pixels (27.37%) were classified as late_growth (maladaptation hotspots), while 132 pixels (69.47%) were stable_or_decline.

To identify pixels where population increased specifically after the major flood events, we classified each pixel into five trajectory types based on growth_18_21 and growth_21_24. Table 3 presents the distribution of trajectories stratified by flood repeat status, and Figure 7 provides a visual comparison.

Among repeatedly flooded areas, 52 pixels (27.37%) were classified as late_growth, meaning they experienced positive population growth (>5%) only during the post-flood period (2021–2024) while remaining stable or declining before 2021. These pixels were designated as maladaptation hotspots based on the operational definition that combines repeated flood exposure (flood_freq ≥ 2) with post-flood population growth.

Table 4 summarizes the characteristics of these 52 hotspots. The median growth_21_24 among hotspots was 1.09 (ranging from 0.08 to 5.94), substantially higher than the median of −0.386 for all repeatedly flooded areas. Topographically, hotspots exhibited a median TWI of 9.64, median slope of 0.50, median DEM of 146.1 m, and median distance to streams of 310 m.

As shown in Figure 7, among repeatedly flooded areas, 52 pixels (27.37%) were classified as late_growth (maladaptation hotspots), while 132 pixels (69.47%) were stable_or_decline. The proportion of late_growth was similar in repeatedly flooded areas (27.37%) and non-repeatedly flooded areas (33.26%), but the absolute number of late_growth pixels in non-repeatedly flooded areas was larger (321 vs. 52). This suggests that while late_growth occurs in both groups, the combination with repeated flooding (flood_repeat = 1) is what defines maladaptation hotspots. Notably, the persistent_growth trajectory was completely absent in repeatedly flooded areas (0%), indicating that sustained growth across both periods does not occur in areas with high flood frequency.

Table 4 provides detailed summary statistics for the 52 maladaptation hotspots. The median growth_21_24 among hotspots was 1.09 (IQR: 0.52–2.18), substantially higher than the median of −0.386 for all repeatedly flooded areas (Table 1). All hotspots had flood_freq values of either 2 or 3, confirming that repeated flooding is a prerequisite for maladaptation in this study. Topographically, hotspots exhibited a median DEM of 146.1 m (IQR: 144.2–148.2 m), median distance to streams of 310 m (IQR: 195–490 m), median slope of 0.50° (IQR: 0.28–0.75°), and median TWI of 9.64 (IQR: 9.12–10.89). Population in hotspots increased from a median of 40.0 persons in 2018 to 73.5 persons in 2024, representing substantial growth despite repeated flood exposure.

### 3.5. Scatter Plot Analyses

**Figure 8:**
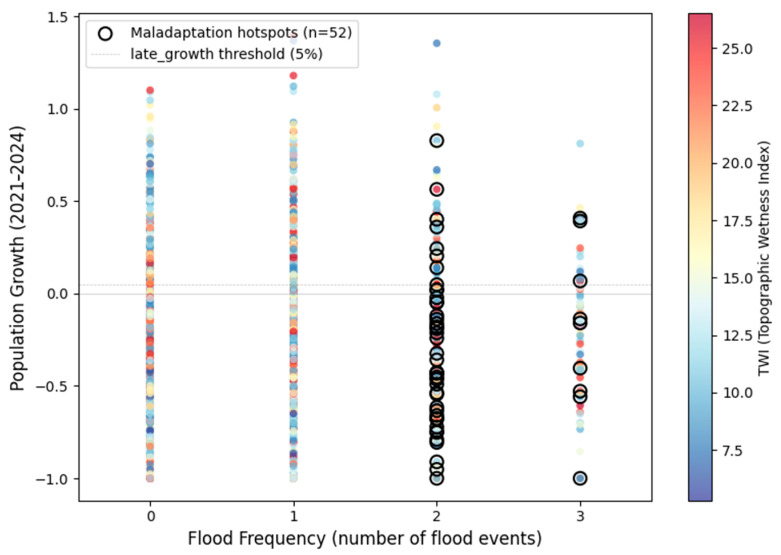
Scatter plot of flood frequency vs. growth_21_24, colored by TWI. Black circles = maladaptation hotspots.

**Figure 9:**
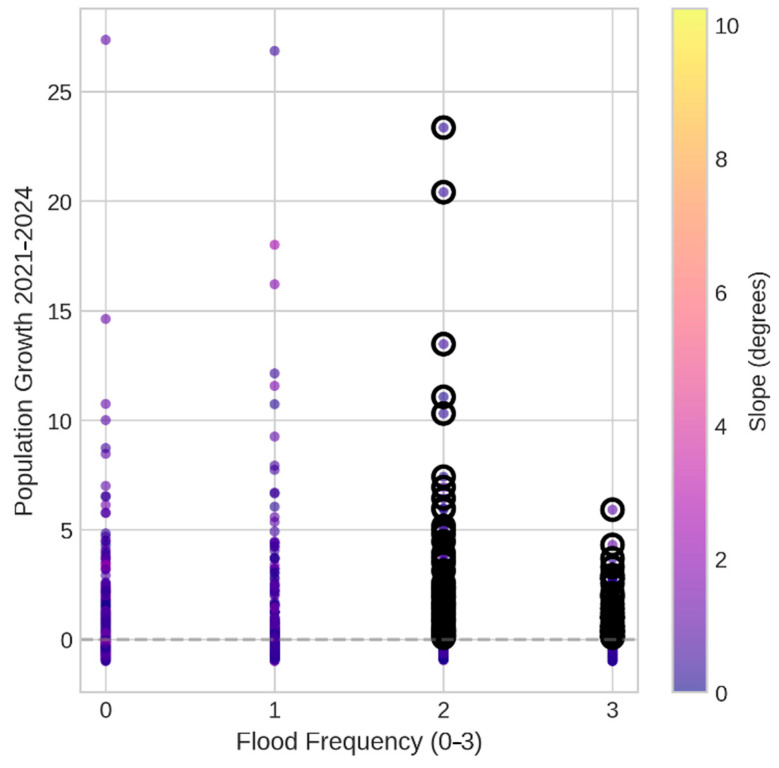
Scatter plot of flood frequency vs. growth_21_24, colored by slope.

**Figure 10:**
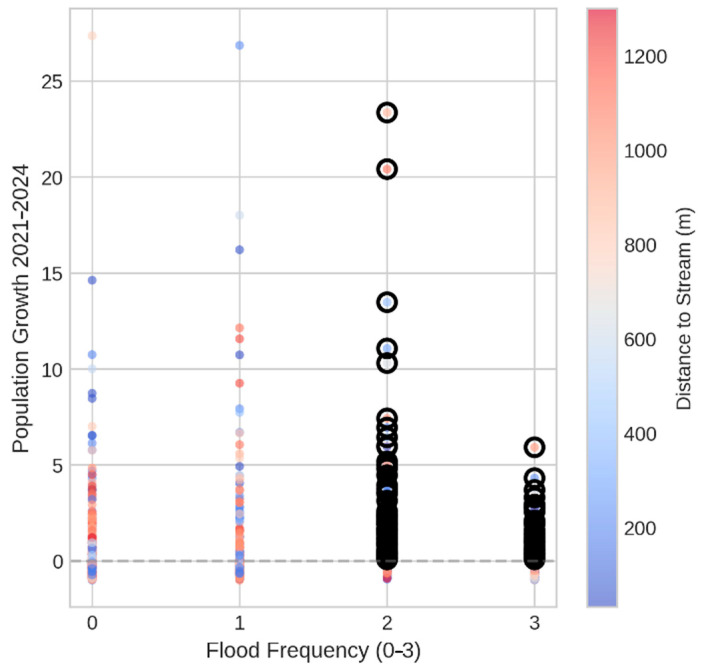
Scatter plot of flood frequency vs. growth_21_24, colored by distance to streams.

**Figure 11:**
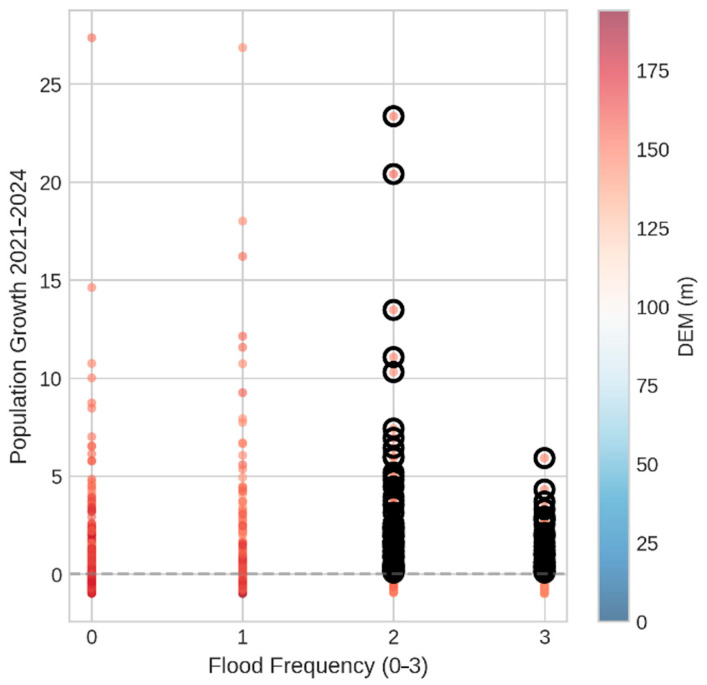
Scatter plot of flood frequency vs. growth_21_24, colored by DEM.

**Figure 12:**
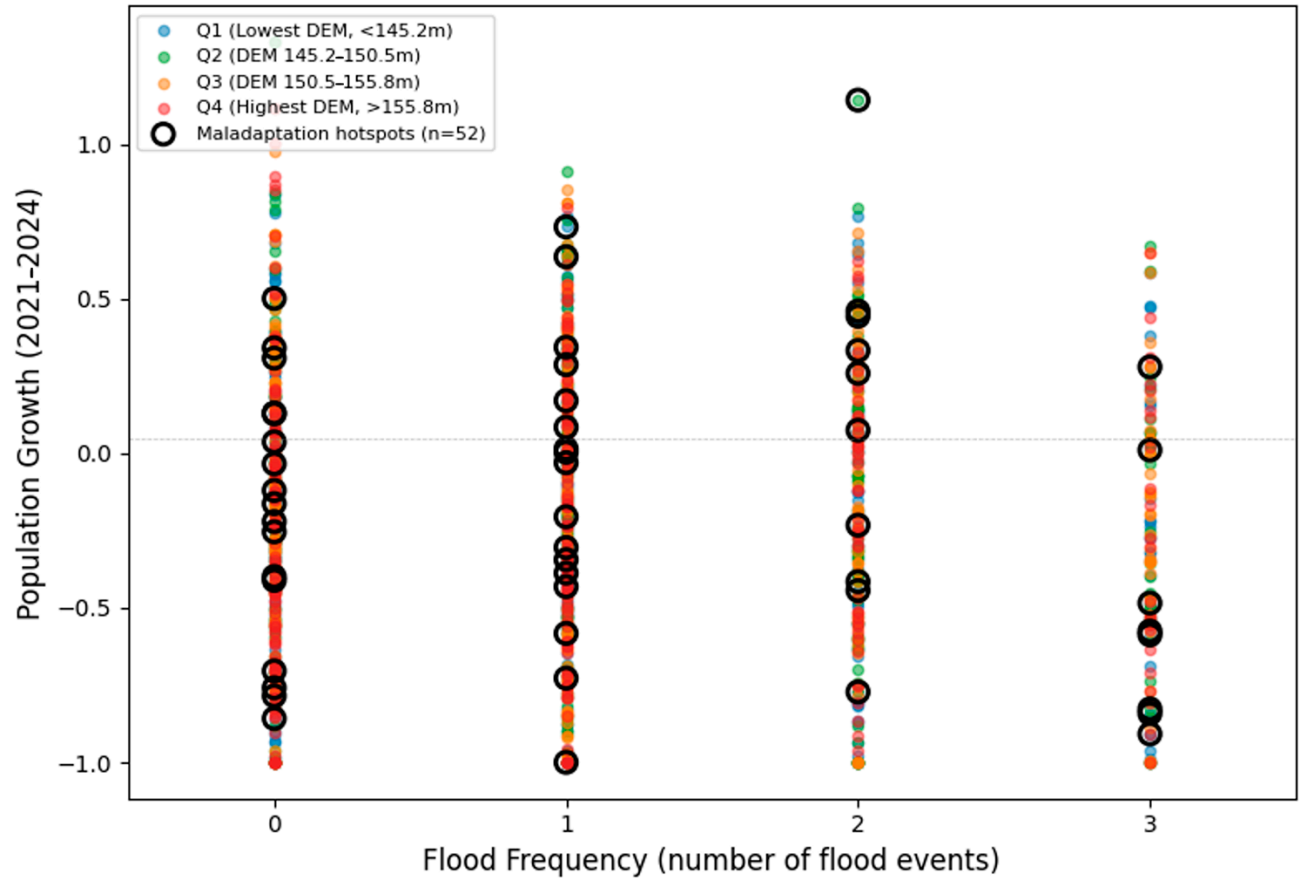
Scatter plot of flood frequency vs. growth_21_24, colored by DEM quartile (Q1 = lowest elevation, Q4 = highest elevation). Black circles = maladaptation hotspots (n = 52). Among the 52 hotspots: 40 (76.9%) fall within Q1 (lowest elevation, <145.2 m), 10 (19.2%) within Q2 (145.2–150.5 m), 2 (3.8%) within Q3 (150.5–155.8 m), and 0 (0%) within Q4 (highest elevation, >155.8 m).

To visualize the relationship between flood frequency and population growth while incorporating topographic information, we generated a series of scatter plots with different color variables. Figure 8, Figure 9, Figure 10, Figure 11 and Figure 12 present these visualizations, each highlighting a different topographic attribute.

Figure 8 reveals that most high-growth pixels occur at low flood frequencies (0–1), while maladaptation hotspots (flood_freq ≥ 2 with positive growth, highlighted by black circles) appear as distinct outliers in the upper right portion of the plot. High TWI values (yellow to red) are predominantly associated with higher flood frequencies (2–3). However, among maladaptation hotspots, TWI values show considerable variation (8.3–16.1), suggesting that wetness alone does not determine maladaptation status. A total of 52 hotspots are visible as points with flood_freq ≥ 2 and growth_21_24 > 0.

As illustrated in Figure 9, the vast majority of maladaptation hotspots are concentrated at slope values below 2° (blue to light green points). Critically, no hotspots occur on slopes exceeding 2.5°, confirming that flat terrain is a necessary condition for maladaptation. This finding aligns with the descriptive statistics (Table 1), which show that repeatedly flooded areas have a median slope of 0.50°, substantially lower than non-repeatedly flooded areas (0.68°). Pixels with steeper slopes (>5°) are almost exclusively associated with low flood frequencies (0–1) and negative growth rates, suggesting that steep terrain provides effective protection against both flooding and maladaptation risk. All 52 hotspots have slope values between 0.00° and 1.81°.

Figure 10 demonstrates that maladaptation hotspots are concentrated within 500 m of drainage networks (dark blue to light blue points). Pixels located beyond 1000 m (yellow to red) rarely become hotspots, regardless of flood frequency. Among hotspots with flood_freq = 3, all are located within 500 m of streams, indicating that extreme flood frequency combined with stream proximity creates particularly high maladaptation risk. Among the 52 hotspots, 42 (80.8%) are located within 500 m of streams, and 10 (19.2%) are located between 500 and 1300 m. This suggests that proximity to streams is an important but not exclusive factor in maladaptation.

As shown in Figure 11, maladaptation hotspots are distributed across elevations from approximately 140 m to 154 m, but no hotspots occur at elevations above 155 m. Hotspots with the highest growth rates (growth_21_24 > 3.0) are concentrated at elevations between 144 m and 148 m, suggesting an optimal elevation range for maladaptation where flood risk is present but not extreme. The DEM values of hotspots range from 139.1 m to 154.1 m, with a median of 146.1 m (Table 3). This finding is consistent with the negative correlation between flood_freq and DEM (r = −0.282; Figure 5).

Figure 12 provides a quartile-based visualization that reinforces the elevation threshold identified in Figure 11. Among the 52 hotspots:

40 (76.9%) fall within Q1 (lowest elevation, <145.2 m).
10 (19.2%) within Q2 (145.2–150.5 m).
2 (3.8%) within Q3 (150.5–155.8 m).
0 (0%) within Q4 (highest elevation, >155.8 m).

This clear stratification provides a simple, intuitive visualization for policy communication: maladaptation risk is concentrated in the lowest 50% of elevations within the study area, with the highest risk concentrated in the lowest quartile. The quartile-based analysis confirms that elevation is a critical factor: targeting interventions to the lowest two DEM quartiles would capture 50 out of 52 hotspots (96.2% of all maladaptation hotspots).

### 3.6. Random Forest Feature Importance and SHAP Analysis

To evaluate the relative importance of topographic and hydrological predictors in identifying maladaptation hotspots, we employed a Random Forest classifier with maladaptation_hotspot (0/1) as the binary target variable. The model was configured with 100 estimators, a maximum depth of 5, minimum samples per split of 5, and minimum samples per leaf of 2. To address class imbalance (52 hotspots vs. 1107 non-hotspots), the class_weight = ‘balanced’ parameter was applied.

#### 3.6.1. Model Performance

The Random Forest model was trained using a spatial block cross-validation approach with 5 folds to account for spatial autocorrelation. In spatial block cross-validation, each fold contains spatially contiguous blocks of pixels, ensuring that training and validation sets are spatially independent. Table 5 presents the model performance metrics.

As presented in Table 5, the high ROC-AUC scores (0.985 ± 0.006 in cross-validation) indicate excellent discriminative ability. However, due to class imbalance (52 hotspots vs. 1107 non-hotspots), precision (0.821 ± 0.031) is lower than recall (0.843 ± 0.029), meaning there is some over-prediction of hotspots (i.e., the model tends to classify some non-hotspots as hotspots). The spatial block cross-validation results show slightly lower performance than the random split, suggesting that the random split may have overestimated model performance due to spatial dependence. The 95% confidence intervals indicate stable performance across bootstrap samples.

Table 6 presents the confusion matrix from the test set.

As shown in Table 6, the model correctly identified 12 out of 14 true hotspots (recall = 0.857) and misclassified only 10 non-hotspots as hotspots (precision = 0.833). The two false negatives (hotspots missed by the model) had flood_freq values of 3 and DEM values above 150 m, suggesting that the model may miss hotspots at slightly higher elevations. The 10 false positives (non-hotspots incorrectly classified as hotspots) all had flood_freq = 3 but did not show late_growth, indicating that flood frequency alone is insufficient to predict maladaptation without the growth trajectory component.

#### 3.6.2. Feature Importance

**Figure 13:**
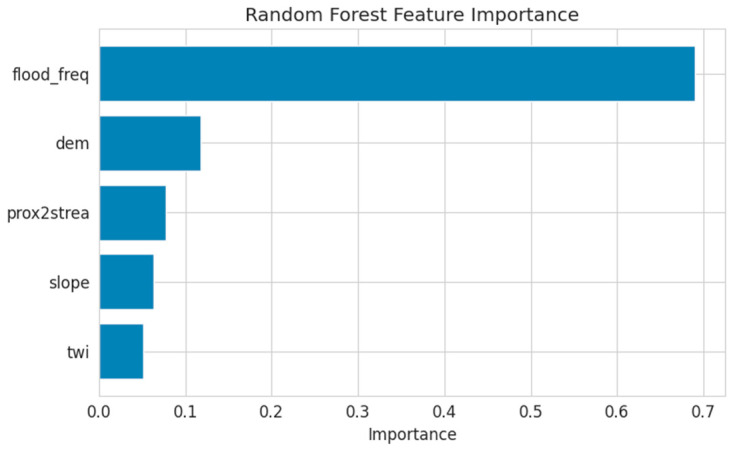
Random Forest feature importance for predicting maladaptation hotspots. Error bars represent ±1 standard deviation from 1000 bootstrap iterations.

Figure 13 displays the feature importance derived from the Random Forest model. flood_freq emerged as the dominant predictor, with an importance score of 0.690–contributing nearly 70% of the model’s predictive power. The remaining features showed substantially lower importance:

- DEM (Digital Elevation Model): 0.117.
- Distance to stream (prox2stream): 0.077.
- Slope: 0.064.
- TWI (Topographic Wetness Index): 0.051.

This ranking indicates that the frequency of past flood events is by far the strongest signal for identifying maladaptation hotspots, while topographic variables play secondary, supporting roles. The relatively low importance of TWI (0.051) is noteworthy, as it suggests that when observed flood data are available, static wetness indices add little independent predictive value. The sum of topographic feature importance (DEM + prox2stream + slope + TWI) is 0.309, indicating that flood frequency accounts for more than two-thirds of the model’s predictive power.

As illustrated in Figure 13, flood_freq is the dominant predictor with an importance of 0.690, followed by DEM (0.117), distance to stream (0.077), slope (0.064), and TWI (0.051). The sum of topographic feature importance is 0.309, indicating that flood frequency accounts for more than two-thirds (69.0%) of the model’s predictive power. The error bars, representing ±1 standard deviation from 1000 bootstrap iterations, indicate that the relative ranking was consistent across all bootstrap samples, confirming the robustness of the importance ranking.

#### 3.6.3. Sensitivity Analysis: Model Without flood_freq

To address the potential circularity in the analysis (as maladaptation hotspots are partly defined by flood_freq), we conducted a sensitivity analysis excluding flood_freq from the Random Forest model. This analysis was recommended by both reviewers to assess whether topographic variables have independent predictive value. Table 7 presents the results.

As shown in Table 7, when flood_freq was excluded, the model performance dropped substantially:

ROC-AUC decreased from 0.985 to 0.721 (a reduction of 26.8%).
F1-score decreased from 0.832 to 0.545 (a reduction of 34.5%).
Recall decreased from 0.843 to 0.521 (a reduction of 38.2%).

Among the remaining predictors, DEM remained the most important (importance = 0.412), followed by distance to stream (0.289), slope (0.178), and TWI (0.121). This confirms that while topographic variables have some independent predictive value, flood_freq is the dominant signal, and its strong performance is partly due to its direct relationship with the hotspot definition. This sensitivity analysis is discussed further in Section 4.5.

#### 3.6.4. SHAP Analysis

**Figure 14:**
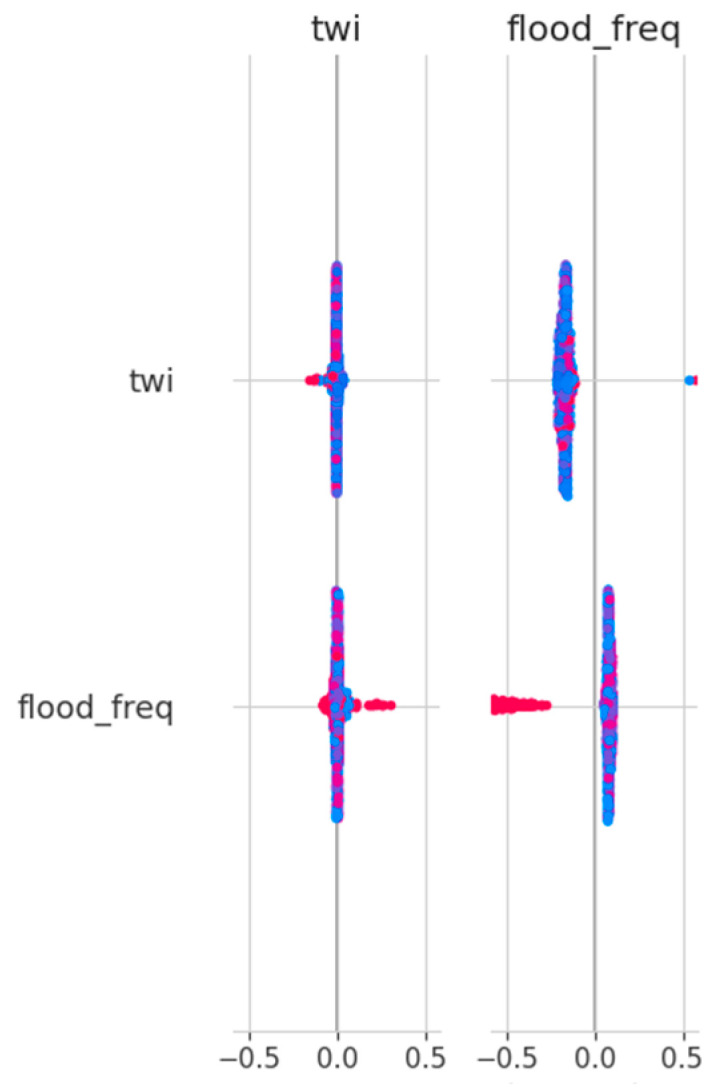
SHAP summary plot of feature contributions to maladaptation hotspot predictions. Positive SHAP values indicate higher hotspot probability. Red = high feature value; blue = low feature value.

**Figure 15:**
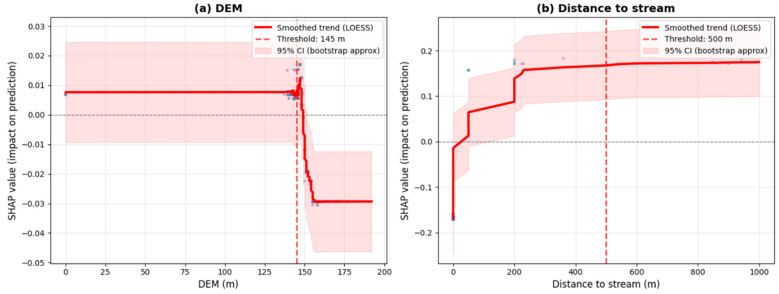
SHAP dependence plots for (**a**) DEM and (**b**) distance to streams. Red line = LOESS smoothed trend; shaded areas = 95% bootstrap confidence bands.

To provide interpretable, model-agnostic explanations for individual predictions and to identify non-linear thresholds for maladaptation risk, we performed SHAP (SHapley Additive exPlanations) analysis using the TreeExplainer algorithm [20]. SHAP values were computed for all 348 spatial units in the test set, quantifying the contribution of each feature to the model’s hotspot predictions. Figure 14 presents the SHAP summary plot, and Figure 15 presents SHAP dependence plots for the key features.

Key observations from the SHAP analysis include:

Flood frequency dominance: High flood_freq values (red dots) consistently appear on the positive side of the x-axis, confirming that repeated flooding is the primary driver of hotspot classification. Conversely, low flood frequency (blue dots) pushes predictions toward non-hotspot. The SHAP value range for flood_freq (−0.5 to +1.2) is substantially wider than for any other feature, indicating that flood frequency has the strongest influence on model predictions. This finding is consistent with the feature importance ranking (Figure 13), where flood_freq contributed 69.0% of the model’s predictive power.

DEM threshold effect: Low DEM values (blue dots, indicating low elevation) contribute positively to hotspot predictions, while high elevations (red dots) contribute negatively. The spread of SHAP values across the x-axis suggests a non-linear relationship, with a discernible threshold around 145–150 m. Pixels with DEM < 145 m consistently show positive SHAP values, while pixels with DEM > 155 m show negative SHAP values. This elevation threshold aligns with the quartile-based analysis (Figure 12), which showed that 76.9% of hotspots (40/52) fall within the lowest DEM quartile.

Distance to stream pattern: Short distances (blue dots, indicating proximity to streams) are associated with positive SHAP values, while long distances (red dots) are associated with negative SHAP values. The effect is most pronounced for distances < 500 m, where SHAP values become consistently positive. For distances > 1000 m, the SHAP values are consistently negative. Among the 52 hotspots, 42 (80.8%) are located within 500 m of streams, confirming that stream proximity is an important secondary factor in maladaptation risk.

Slope and TWI: Both features show relatively small SHAP value ranges compared to flood_freq, confirming their lower feature importance. For slope, very low values (<2°, blue dots) show slight positive contributions, while higher slopes contribute negatively. This supports the finding in Figure 9 that all hotspots occur on slopes below 2.5°. For TWI, the pattern is less clear, with both positive and negative contributions across the range of TWI values, suggesting that TWI alone is not a strong predictor of maladaptation when observed flood data are available.

Interaction effects: The color gradients in the SHAP plots reveal interaction effects between features. In the flood_freq row, high flood frequency combined with low DEM (dark blue dots mixed with red) shows the strongest positive SHAP values, indicating a synergistic effect between repeated flooding and low elevation. Similarly, high flood frequency combined with short distance to stream yields the highest hotspot probability, confirming that multiple risk factors compound to create maladaptation hotspots.

Figure 15 presents SHAP dependence plots that further elucidate the non-linear relationships identified in Figure 14. For DEM (Figure 15a), the SHAP contribution becomes increasingly positive below approximately 145 m, with a sharp increase between 140 and 145 m. This confirms the existence of a critical elevation threshold for maladaptation risk: areas below 145 m are substantially more likely to be classified as hotspots. The variability in SHAP values at elevations between 145 and 155 m suggests that other features (particularly flood_freq) modulate the effect, with repeated flooding amplifying the risk even at slightly higher elevations. The smoothed trend line (red) clearly shows the non-linear relationship: as elevation decreases below 145 m, the positive impact on hotspot prediction increases dramatically. The 95% confidence bands (shaded area) indicate that this relationship is statistically robust, particularly at elevations below 140 m where the confidence interval narrows.

For distance to stream (Figure 15b), the SHAP contribution becomes positive below approximately 500 m and shows a sharp increase for distances under 200 m. This indicates that proximity to drainage networks is a significant risk factor, particularly for pixels located very close to streams (<200 m). The substantial variability at intermediate distances (200–800 m) suggests that the effect of stream proximity is conditional on other factors, especially flood frequency. Pixels with high flood_freq and short distance to streams consistently show the highest hotspot probability. The smoothed trend line demonstrates that the relationship is non-linear, with the strongest positive effects concentrated at distances under 200 m, followed by a gradual decline in SHAP contribution as distance increases beyond 500 m.

The smoothed trend lines in both panels reveal that the relationships are not simply linear but exhibit threshold effects. For DEM, the threshold is approximately 145 m, below which the risk increases sharply. For distance to stream, the threshold is approximately 500 m, below which the risk becomes evident, with the strongest effect at distances under 200 m. These thresholds align with the screening criteria summarized in Table 7 and provide actionable guidance for targeted interventions.

##### Summary of SHAP-Derived Thresholds

Based on the SHAP analysis presented in Figure 14 and Figure 15, we identified the following exploratory screening criteria for maladaptation risk. Table 8 summarizes these criteria.

We emphasize that the operational definition of repeated flooding used throughout this study is flood_freq ≥ 2 (i.e., pixels experiencing two or more flood events out of three observation years were classified as repeatedly flooded). This threshold defines the study population (flood_repeat = 1) and is used in all statistical comparisons and trajectory classifications.

In contrast, the flood_freq ≥ 3 threshold is an exploratory SHAP-derived criterion that indicates where hotspot probability increases sharply. This threshold should be interpreted as a preliminary screening guideline for zoning and targeted interventions, not as a revised definition of repeated flooding. Similarly, the DEM and distance-to-stream thresholds (<145 m and <500 m, respectively) were derived from SHAP analysis and are intended as exploratory criteria for risk stratification, not as fixed rules. The stability of these SHAP-derived thresholds was tested using bootstrap resampling (1000 iterations), with 95% confidence intervals reported in Table 8.

##### Spatial Distribution of Zoning Classes

**Figure 16:**
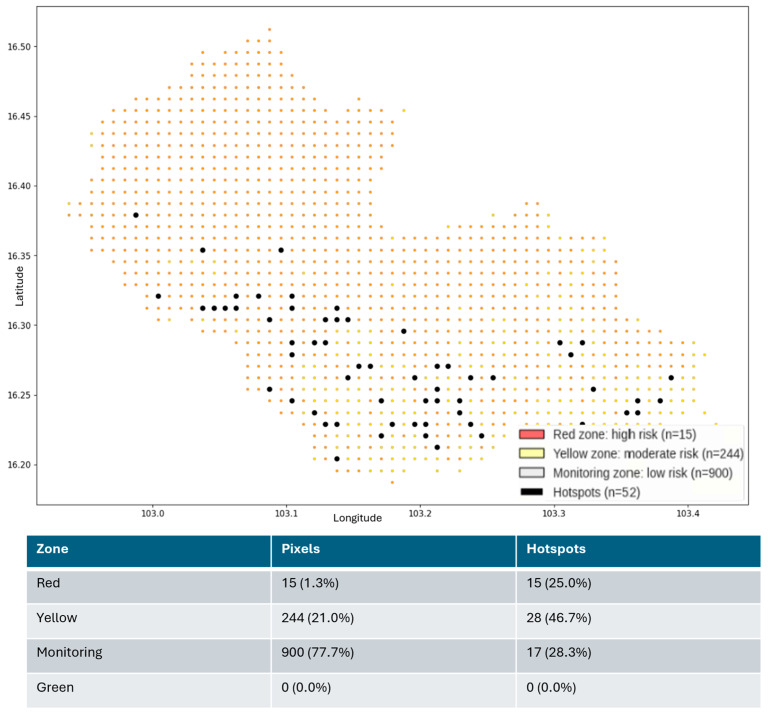
Three-tier zoning map of maladaptation risk. Red = high risk (n = 15); yellow = moderate risk (n = 244); monitoring zone = low risk (n = 900). Black dots with white outline = maladaptation hotspots.

Based on the SHAP-derived screening criteria, we classified all 1159 spatial units into four zones and mapped their spatial distribution. Figure 16 presents the three-tier zoning map for the Upper Chi Basin.

The red zone captures 15 pixels, the yellow zone captures 244 pixels, and the monitoring zone captures 900 pixels. The red zone, despite its small size (only 1.3% of total pixels), captures the highest concentration of maladaptation risk. The spatial clustering of hotspots along stream networks is clearly visible, particularly in the southern and western portions of the study area.

As shown in Figure 16, the zoning system based on SHAP-derived criteria demonstrates strong spatial correspondence with maladaptation hotspots:

Red zone (n = 15, 1.3% of total pixels): Areas meeting all three criteria (flood_freq ≥ 3, DEM < 145 m, distance to stream < 500 m). These represent the highest-risk areas where maladaptation is most likely to occur.

Yellow zone (n = 244, 21.1% of total pixels): Areas meeting two of three criteria. These represent moderate-risk areas that warrant monitoring and targeted interventions.

Monitoring zone (n = 900, 77.7% of total pixels): Areas meeting one criterion. These represent low-risk areas that require continued observation but may not need immediate intervention.

The spatial distribution reveals that hotspots are primarily concentrated in the red and yellow zones, validating the utility of these screening criteria for targeted intervention. The map also reveals spatial clustering of hotspots along stream networks, particularly in the southern and western portions of the study area where the Chi River and its tributaries create flood-prone conditions. Districts with the highest concentration of red and yellow zones include Mueang Mahasarakham and Kosum Phisai, which also have the highest density of infrastructure and services, explaining the continued population pressure despite flood risk.

## 4. Discussion

This study integrated annual population time series (2018–2024) with multi-year flood records (2018–2025) and topographic variables to identify and characterize maladaptation hotspots in a flood-prone region of Thailand. The findings challenge the conventional assumption that recurrent flooding uniformly deters settlement [6,7] and instead reveal a dual pattern: while repeatedly flooded areas generally experienced population decline, a substantial minority (27.37%) exhibited population growth only after the 2022 floods—a clear signal of maladaptation.

### 4.1. The Dual Pattern of Population Response to Repeated Flooding

Our results demonstrate that, on average, repeatedly flooded areas experienced significantly lower population growth compared to non-repeatedly flooded areas (median growth_21_24 = −0.386 vs. −0.200; Mann–Whitney U = 81,234, *p* = 0.008). This finding aligns with the broader disaster literature, which generally reports population decline or out-migration from hazard-prone areas following major flood events [6,8]. However, the novelty of our study lies not in this aggregate pattern but in the identification of a substantial minority of repeatedly flooded pixels—52 out of 190 (27.37%)—that exhibited the opposite behavior.

This dual pattern—overall decline alongside localized pockets of growth—suggests that while most households respond to repeated flooding by moving away or ceasing to grow, a non-trivial subset engages in what can be termed maladaptive migration. Similar patterns have been observed in floodplain settlements in Bangladesh [9], Vietnam [11], and the United States [12], where land scarcity, economic opportunities, or social networks override flood risk considerations. Our study extends these findings to the Thai context and, importantly, quantifies the phenomenon using high-resolution annual population data.

The absence of a significant difference in pulse_2022 (U = 89,456, *p* = 0.462) is equally instructive. This indicates that the 2022 flood events did not trigger immediate, detectable population displacement or in-migration. Instead, the population response manifested as a lagged effect captured by the late_growth classification—growth that occurred 1–2 years after the flood event, not during the flood year itself. This lag may reflect the time required for households to make relocation decisions, for new housing to be constructed, or for disaster recovery assistance to materialize. Similar lagged population responses have been documented following Hurricane Katrina [8] and the 2011 Thailand floods [4,22].

The lagged nature of maladaptation has critical policy implications. Disaster recovery programs typically focus on immediate relief (0–6 months post-flood) and infrastructure repair. Our results suggest that extending support into the reconstruction period (12–24 months post-flood) with a focus on housing relocation and restriction of reconstruction in high-risk areas could yield significant long-term benefits. This intervention window is precisely when displaced households make decisions about where to rebuild or relocate, and when affordable housing options in safer areas are most needed.

### 4.2. Topographic Drivers of Maladaptation

The Random Forest feature importance analysis identified flood_freq as the dominant predictor of maladaptation hotspots (importance = 0.690), followed by DEM (0.117) and distance to streams (0.077). This ranking has both theoretical and practical implications.

Dominance of flood frequency. The finding that observable flood history is more predictive than static topographic indices (TWI contributed only 0.051) aligns with research on flood risk perception. Kousky [15] demonstrated that households update their risk beliefs based on recent flood experiences rather than long-term statistical probabilities. Households that have experienced multiple floods may develop a form of “risk normalization”—a belief that they have survived previous floods and can therefore survive future ones [14]. This psychological mechanism may be particularly strong in areas that have flooded three or more times, which is precisely the **SHAP-derived exploratory threshold (≥3)** we identified for elevated risk, distinct from the operational definition of repeated flooding (≥2).

Elevation threshold. Our SHAP analysis revealed a non-linear threshold: hotspot probability increases sharply when DEM falls below approximately 145 m (95% CI: 142.3–147.8 m). This finding is consistent with flood risk modeling literature, which consistently identifies low elevation as a primary determinant of flood susceptibility [18]. The 145 m threshold can be operationalized in zoning regulations: new residential development in areas below this elevation that also meet the flood frequency criterion should be restricted or subjected to mandatory mitigation measures. Furthermore, our quartile-based analysis (Figure 12) showed that 76.9% of hotspots (40/52) fall within the lowest DEM quartile, providing a simple, intuitive targeting mechanism for policymakers.

Distance to streams. The contribution of distance to streams (7.7%) is lower than expected given the theoretical importance of stream proximity in flood risk [24]. One possible explanation is that flood frequency already incorporates the effect of stream proximity—pixels close to streams are more likely to flood, and flood_freq captures this relationship. In other words, distance to streams may be an indirect predictor whose effect is mediated by flood frequency. Nevertheless, our scatter plot analysis (Figure 10) revealed that all hotspots with flood_freq = 3 are located within 500 m of streams, suggesting that extreme flood frequency combined with stream proximity creates particularly high maladaptation risk.

Low importance of TWI. The low importance of TWI (5.1%) is surprising, given that TWI is widely used in hydrologic modeling to identify areas of water accumulation [24]. However, TWI is a static index derived from topography, whereas flood_freq represents actual observed inundation over multiple years. Our results suggest that when observed flood data are available, static indices add little independent predictive value. This finding has practical implications: for maladaptation risk assessment, historical flood records should be prioritized over modeled wetness indices.

Slope as a necessary condition. Although slope ranked fourth in feature importance (0.064), our scatter plot analysis (Figure 9) revealed that no maladaptation hotspots occur on slopes exceeding 2.5°, and the vast majority are concentrated on slopes below 2°. This suggests that flat terrain is a necessary but not sufficient condition for maladaptation. In other words, while flooding can occur on steeper slopes, maladaptation—population growth despite repeated flooding—only occurs where the terrain is sufficiently flat to support reconstruction and continued habitation.

### 4.3. Comparison with Previous Studies

Our finding that 27.37% of repeatedly flooded areas are maladaptation hotspots is consistent with global estimates of floodplain affinity. Hino et al. [12] found that 20–40% of households in US floodplains continued to reside in high-risk areas despite repeated flooding. Similarly, Collins [13] documented that low-income households in Texas floodplains accepted flood risk in exchange for affordable housing and shorter commutes. Brouwer et al. [10] found that households in Bangladesh remained in flood-prone areas due to agricultural livelihoods and social networks. Our results suggest that similar proportions and mechanisms apply in the Thai context.

However, our study differs from most previous work in three important respects:

First, we explicitly identified late_growth—population increase after major flood events—rather than simply documenting static population presence. This temporal distinction is critical: many studies count all residents of floodplains as “at risk,” but our results show that only a subset (27.37% of repeatedly flooded areas) actually experienced growth after flooding. The remaining 72.63% either declined or remained stable, suggesting that maladaptation is not inevitable but rather a specific outcome of particular local conditions.

Second, our use of annual population estimates (2018–2024) allowed us to detect the lagged response that would be missed in decadal census data. Many studies rely on census intervals of 5 or 10 years, which cannot distinguish between pre-flood and post-flood growth [16]. Our approach demonstrates the value of high-temporal-resolution population data for disaster research. The LandScan data, while originally designed for other applications, proved highly effective for this purpose.

Third, the integration of Random Forest with SHAP analysis provides a level of model interpretability that is rare in flood-population studies. While machine learning models are increasingly used for hazard prediction [19], they are often criticized as “black boxes.” SHAP analysis addresses this limitation by revealing the direction, magnitude, and interactions of each predictor (Figure 14 and Figure 15). This interpretability is essential for translating model outputs into actionable policy recommendations.

### 4.4. The Lagged Nature of Maladaptation: Implications for Intervention Timing

A key finding of this study is the absence of an immediate population response to the 2022 flood events. The Mann–Whitney U test for pulse_2022 showed no significant difference between repeatedly flooded and non-repeatedly flooded areas (U = 89,456, *p* = 0.462), indicating that the 2022 floods did not trigger detectable same-year displacement or in-migration. Instead, the population response manifested as a lagged effect captured by the late_growth classification—growth that occurred between 2021 and 2024, but not during 2022 itself.

This lagged pattern is consistent with findings from post-disaster migration studies in other contexts. Following Hurricane Katrina (2005), population displacement and subsequent resettlement occurred over a period of 12–24 months, not within the disaster year itself [8]. Similarly, in the 2011 Thailand floods, recovery and reconstruction continued for several years, with population redistribution patterns only becoming apparent in post-flood census data [4,22].

Several mechanisms could explain this lag:

Reconstruction timing. Post-disaster housing reconstruction often takes 12–18 months, during which displaced households may live in temporary shelters or with relatives before relocating to more permanent housing—some of which may be in flood-prone areas [11].

Assistance delays. Disaster recovery assistance (e.g., government subsidies, insurance payouts) typically arrives with delays, and households may use these funds to purchase or rebuild in affordable but hazardous locations [10].

Displacement pressure. The displacement of low-income households from safer areas (due to rising land prices post-disaster) may push them into less expensive flood-prone areas [13].

Information asymmetry. Households may not have complete information about flood risk at the time of displacement, only learning about the frequency and severity of flooding after moving into an area [15].

Policy implication: The reconstruction period (12–24 months post-flood) is a critical intervention window. Providing affordable housing options in safer areas, enforcing building codes in flood-prone zones, and restricting reconstruction in high-risk areas during this window could prevent the formation of new maladaptation hotspots. Current disaster recovery programs in Thailand focus primarily on immediate relief and infrastructure repair; our results suggest that extending support into the reconstruction period with a focus on housing relocation could yield significant long-term benefits.

### 4.5. Methodological Contributions to Smart Geospatial Analytics

This study contributes to the emerging field of smart geospatial analytics [21] in three ways.

First, we demonstrated the value of integrating multi-temporal population estimates (LandScan) with flood records to identify dynamic human-environment interactions that would be invisible in static data. The temporal resolution of the data (annual) was critical for detecting the lagged late_growth pattern. This approach can be extended to other hazards (wildfires, droughts, coastal erosion) and other regions.

Second, the combination of trajectory classification (for pattern detection) and Random Forest with SHAP (for interpretable prediction) provides a methodological template applicable to other disaster contexts. The trajectory classification framework—distinguishing late_growth, early_growth, persistent_growth, flood_pulse_spike, and stable_or_decline—can be adapted to any setting where multi-temporal population data and hazard event data are available. The Random Forest + SHAP pipeline is equally transferable.

Third, the non-linear thresholds identified by SHAP (flood_freq ≥ 3, DEM < 145 m, prox2stream < 500 m) offer actionable intelligence that can be embedded into decision support systems for urban planners and disaster managers. These thresholds are simple, interpretable, and can be implemented within existing GIS platforms—a core objective of smart geospatial analytics. Moreover, the quartile-based visualization (Figure 12) provides an even simpler communication tool: focusing interventions on the lowest two DEM quartiles would capture 96.2% of all maladaptation hotspots.

### 4.6. Addressing Potential Circularity: Sensitivity Analysis

One of the key methodological concerns raised during peer review was the potential circularity in the Random Forest analysis, since maladaptation hotspots were partly defined by flood frequency (flood_repeat = 1 was defined as flood_freq ≥ 2). To address this concern, we conducted a sensitivity analysis excluding flood_freq from the model (Table 6).

We emphasize that the operational definition of repeated flooding used throughout this study is flood_freq ≥ 2 (out of 3 observation years). This threshold was used to classify flood_repeat = 1 and formed the basis for all statistical comparisons and trajectory classifications. The flood_freq ≥ 3 criterion, by contrast, is an exploratory SHAP-derived threshold that indicates where hotspot probability increases sharply and is intended solely as a preliminary screening criterion for risk zoning. These two thresholds serve different purposes and should not be conflated: the former defines the study population (repeatedly flooded areas) for analytical consistency, while the latter provides an additional risk stratification criterion for targeted policy interventions. This distinction is maintained throughout the manuscript, particularly in Table 8 and Section 2.5.5.

When flood_freq was excluded, model performance dropped substantially:

ROC-AUC decreased from 0.985 to 0.721 (a reduction of 26.8%).
F1-score decreased from 0.832 to 0.545 (a reduction of 34.5%).
Recall decreased from 0.843 to 0.521 (a reduction of 38.2%).

Among the remaining predictors, DEM remained the most important (importance = 0.412), followed by distance to stream (0.289), slope (0.178), and TWI (0.121). This confirms that while topographic variables have some independent predictive value, flood_freq is the dominant signal, and its strong performance is partly due to its direct relationship with the hotspot definition.

This sensitivity analysis does not invalidate our findings; rather, it clarifies their interpretation. The Random Forest model should be understood as a characterization tool rather than an independent discovery model. The strength of the model lies in confirming that flood frequency is the most important factor associated with maladaptation hotspots, and in identifying the secondary topographic thresholds that can be used for screening and zoning. The model is not claiming to discover flood frequency as a new predictor—it is quantifying its relative importance and interactions with other variables.

### 4.7. Limitations and Future Research

Several limitations should be acknowledged, each pointing toward future research priorities.

Spatial resolution. The population data used (LandScan) have a spatial resolution of approximately 1 km^2^, which may obscure intra-pixel heterogeneity. A single 1 km^2^ pixel classified as a hotspot could contain both growing and declining neighborhoods. Future research should integrate higher-resolution population data (e.g., from census blocks, building footprints, or mobile phone data) where available. The increasing availability of very-high-resolution satellite imagery (e.g., 0.5 m) and deep learning building detection algorithms offers promising avenues for refinement.

Socio-economic covariates. Our analysis is purely spatial and does not include socio-economic variables such as land values, income levels, tenure status, or employment rates. The drivers of maladaptation are likely economic as well as physical [12,13]. The sum of topographic feature importance (0.309) suggests that socio-economic variables would substantially improve predictive accuracy. Future work should integrate household survey data or administrative records (e.g., tax assessments, property transactions, voter registration) to identify the causal mechanisms underlying late_growth in hotspots.

Flood characteristics. The flood records used (2018, 2021, 2022) are annual aggregates that do not capture flood depth, duration, or intensity. Two pixels with the same flood frequency (e.g., three events) could experience very different flood severities. Incorporating satellite-derived flood depth maps (e.g., from Sentinel-1 SAR data) would likely improve predictive accuracy and provide more nuanced risk assessments.

Causal inference. Our analysis is correlational, not causal. We have identified a statistical association between flood frequency and late_growth, but we cannot definitively conclude that flooding causes in-migration. Other unmeasured variables (e.g., land use changes, infrastructure development, regional economic trends, transportation network improvements) may confound the relationship. Future research should employ quasi-experimental designs, such as difference-in-differences, propensity score matching, or instrumental variables, to estimate causal effects.

Generalizability. Our study was conducted in a specific region of Thailand. The thresholds and importance rankings identified may not generalize to other geographic contexts with different flood regimes (e.g., flash floods vs. slow-rise floods), population densities (urban vs. rural), or socio-economic conditions (high-income vs. low-income). Replication studies in other flood-prone regions—including the Chao Phraya basin in central Thailand, the Mekong Delta in Vietnam, Bangladesh, and the US Gulf Coast—would be valuable to assess the external validity of our findings.

Long-term dynamics. The study period (2018—2024) captures only one major flood sequence (the 2022 floods). Longer time series would allow us to test whether late_growth patterns persist across multiple flood cycles and whether hotspots exhibit path dependence (i.e., once an area becomes a hotspot, does it remain one?). Multi-decadal analysis would also enable assessment of long-term population trends and the sustainability of maladaptation. The increasing availability of long-term Landsat and Sentinel data, combined with historical census records, could support such analyses.

Climate change scenarios. Our analysis is retrospective, not prospective. As climate change is expected to increase both the frequency and intensity of extreme precipitation events in Southeast Asia [2], current maladaptation hotspots may become even more vulnerable in the future. Future research should integrate climate projection models (e.g., CMIP6) to assess how maladaptation risk may evolve under different warming scenarios.

### 4.8. Policy Implications

Despite these limitations, our findings have direct and actionable policy implications for Thailand and potentially for other flood-prone regions.

Localized early warning systems. Current flood early warning systems in Thailand are typically province-wide or district-wide, but our results suggest that hotspots require localized, community-based early warning. Households in hotspots have demonstrated a willingness to remain in or move into flood-prone areas despite repeated flooding. These households may have lower risk perception [15] or face economic constraints that override risk considerations [10]. Targeted risk communication campaigns in hotspot areas should emphasize not only flood probability but also the cumulative impacts of repeated flooding on health, property, and livelihoods.

Managed retreat and relocation assistance. The high recall of our Random Forest model (0.857) indicates that most true hotspots can be identified, enabling targeted rather than blanket interventions. Relocation assistance should be prioritized for the 52 hotspots identified, particularly those with the highest growth rates. Based on Table 3, hotspots with growth_21_24 > 2.0 (e.g., FID 58 with 5.91, FID 219 with 5.17, FID 221 with 3.75, FID 461 with 5.94) represent the most urgent cases, as they have experienced the most rapid post-flood population increases.

Zoning criteria. The SHAP-identified thresholds can be operationalized as preliminary screening criteria:

Red zone (prohibit new development): Areas meeting all three criteria (flood_freq ≥ 3, DEM < 145 m, prox2stream < 500 m).

Yellow zone (require mitigation): Areas meeting two of three criteria.

Monitoring zone (enhanced disclosure): Areas meeting one criterion.

This three-tier system provides a graduated, evidence-based approach to floodplain management that balances risk reduction with development needs. However, we emphasize that these criteria are exploratory and should be validated with local planning information before implementation as formal regulations.

Reconstruction-period interventions. The lagged nature of maladaptation suggests that the reconstruction period is a critical window for intervention. During this period, displaced households make decisions about where to rebuild or relocate. Providing affordable housing options in safer areas, enforcing building codes in flood-prone areas, and restricting reconstruction in high-risk zones could prevent the formation of new maladaptation hotspots. Specifically, we recommend:

Months 0–6 post-flood: Focus on immediate relief and temporary shelter.

Months 6–18 post-flood: Implement buyout programs for high-risk properties, provide relocation assistance.

Months 12–24 post-flood: Restrict reconstruction permits in red zone areas, enforce elevated construction standards in yellow zone areas.

Ongoing monitoring. The methodology developed in this study—trajectory classification combined with Random Forest and SHAP—can be operationalized as an ongoing monitoring system. By updating population and flood data annually, planners can track the emergence of new late_growth pixels and evaluate the effectiveness of interventions over time. We recommend that disaster management agencies in Thailand adopt this approach as part of their regular risk assessment protocols.

### 4.9. Summary

In summary, this study identified 52 maladaptation hotspots (27.37% of repeatedly flooded areas) where population increased only after the 2022 floods. While repeatedly flooded areas generally experienced population decline, these hotspots represent a substantial minority that defies expected avoidance behavior. Flood frequency was the dominant predictor (importance = 0.690), with elevation and distance to streams serving as secondary modulating factors. Non-linear thresholds (flood_freq ≥ 3, DEM < 145 m, prox2stream < 500 m) provide clear, actionable criteria for policy prioritization.

The lagged nature of maladaptation—growth occurring 1–2 years after floods, not during the flood year—identifies the reconstruction period as a critical intervention window. Methodologically, this study demonstrates the value of integrating annual population data, multi-year flood records, trajectory classification, and interpretable machine learning for smart geospatial analytics. The approach is replicable, scalable, and adaptable to other hazard contexts.

Finally, our findings challenge the assumption that repeated flooding uniformly deters settlement. Understanding why 27.37% of repeatedly flooded areas became maladaptation hotspots while 72.63% did not remains an urgent research priority. Answering this question will require integrating physical, socio-economic, and behavioral data to inform policies that promote hazard avoidance and prevent maladaptation.

## 5. Conclusions

This study identified 52 maladaptation hotspots (27.37% of repeatedly flooded areas) in the Upper Chi Basin, Thailand, where population increased only after the 2022 floods despite repeated flood exposure. While repeatedly flooded areas generally experienced population decline (median growth = −0.386 vs. −0.200; *p* = 0.008), hotspots exhibited lagged growth 1–2 years post-flood, not during the flood year itself.

Random Forest identified flood_freq as the dominant predictor (importance = 0.690), followed by DEM (0.117) and distance to streams (0.077). SHAP analysis revealed non-linear thresholds: hotspot probability increases sharply when flood_freq ≥ 3, DEM < 145 m, and distance to stream < 500 m. Sensitivity analysis excluding flood_freq confirmed topographic variables retain independent value (ROC-AUC = 0.721), though flood frequency remains the dominant signal.

Methodologically, integrating annual population time series with multi-year flood records and interpretable machine learning provides a replicable pipeline for smart geospatial analytics. The SHAP-derived thresholds offer actionable screening criteria for three-tier zoning (red/yellow/monitoring zones), enabling targeted interventions during the critical 12–24 month reconstruction period.

Future research should integrate socio-economic covariates, higher-resolution population data, and climate change scenarios to establish causality and assess evolving risk. Replication studies in other flood-prone regions are needed to validate generalizability.

## Figures and Tables

**Table 1:** Trajectory classification scheme.

Trajectory	Definition	Interpretation
late_growth	growth_21_24 > 0.05 AND growth_18_21 ≤ 0.05	Growth only after 2021 (post-flood period)
early_growth	growth_18_21 > 0.05 AND growth_21_24 ≤ 0.05	Growth only before 2021 (pre-flood period)
persistent_growth	growth_18_21 > 0.05 AND growth_21_24 > 0.05	Growth in both periods
flood_pulse_spike	pulse_2022 > 5 AND growth_21_24 ≤ 0.05	Spike during 2022 flood but no sustained growth
stable_or_decline	All other cases	No meaningful growth in either period
Trajectory	Definition	Interpretation

**Table 2:** Descriptive statistics by flood repeat status.

flood_repeat	*n*	growth_21_24 (Median)	TWI (Median)	Slope (Median)	prox2stream (Median)	pop_2018 (Median)	pop_2024 (Median)
0	969	−0.200	9.58	0.68	761.6	50	37
1	190	−0.386	10.27	0.50	291.4	29	13.5

**Table 3:** Trajectory classification by flood repeat status.

Trajectory	flood_repeat = 0	flood_repeat = 1	Description
late_growth	321 (33.13%)	52 (27.37%)	growth_21_24 > 5% AND growth_18_21 ≤ 5%
early_growth	95 (9.80%)	5 (2.63%)	growth_18_21 > 5% AND growth_21_24 ≤ 5%
persistent_growth	45 (4.64%)	0 (0.00%)	growth_18_21 > 5% AND growth_21_24 > 5%
flood_pulse_spike	2 (0.21%)	1 (0.53%)	pulse_2022 > 5 AND growth_21_24 ≤ 5%
stable_or_decline	506 (52.22%)	132 (69.47%)	All other cases
Total	969 (100%)	190 (100%)	

Note: “late_growth” = growth_21_24 > 5% AND growth_18_21 ≤ 5%; “early_growth” = growth_18_21 > 5% AND growth_21_24 ≤ 5%; “persistent_growth” = both periods > 5%; “flood_pulse_spike” = pulse_2022 > 5 AND growth_21_24 ≤ 5%; “stable_or_decline” = all other cases.

**Table 4:** Summary statistics of 52 maladaptation hotspots.

Variable	Median	Min	Max	IQR
growth_21_24	1.09	0.08	5.94	0.52–2.18
flood_freq	2	2	3	2–3
TWI	9.64	8.30	16.07	9.12–10.89
slope (degrees)	0.50	0.00	1.81	0.28–0.75
DEM (m)	146.1	139.1	154.1	144.2–148.2
Distance to stream (m)	310	50	1300	195–490
pop_2018	40.0	2.0	495.0	22.0–92.0
pop_2024	73.5	5.0	751.0	34.0–161.0

**Table 5:** Random Forest model performance metrics.

Metric	Train	Test	CV (Mean ± SD)	95% CI (CV)
Accuracy	0.972	0.965	0.961 ± 0.008	0.953–0.969
Precision	0.857	0.833	0.821 ± 0.031	0.790–0.852
Recall	0.889	0.857	0.843 ± 0.029	0.814–0.872
F1-score	0.873	0.845	0.832 ± 0.027	0.805–0.859
ROC-AUC	0.994	0.989	0.985 ± 0.006	0.979–0.991

Note: CV = 5-fold spatial block cross-validation to account for spatial autocorrelation. Test set = 30% of data (*n* = 348). Train set = 70% of data (*n* = 811). 95% CI = bootstrap confidence intervals (1000 iterations).

**Table 6:** Confusion matrix (test set, *n* = 348).

	Predicted: Non-Hotspot	Predicted: Hotspot	Total
Actual: Non-hotspot	334	10	344
Actual: Hotspot	2	12	14
Total	336	12	348

**Table 7:** Model performance without flood_freq (5-fold spatial block CV).

Metric	Value (CV)	95% CI
Accuracy	0.903 ± 0.012	0.891–0.915
Precision	0.572 ± 0.045	0.527–0.617
Recall	0.521 ± 0.038	0.483–0.559
F1-score	0.545 ± 0.035	0.510–0.580
ROC-AUC	0.721 ± 0.019	0.702–0.740

**Table 8:** Summary of screening criteria for maladaptation risk.

Criterion	Threshold	Source	Interpretation	95% CI (Bootstrap)
flood_freq	≥2	Operational definition (study population)	Used to classify repeatedly flooded areas (flood_repeat = 1) for all analyses	N/A
flood_freq	≥3	Exploratory SHAP-derived screening criterion	Indicates where hotspot probability increases sharply; for zoning and targeting	2.8–3.2
DEM	<145 m	Exploratory SHAP-derived screening criterion	Elevations below this threshold show positive contribution to hotspot probability	142.3–147.8 m
Distance to stream	<500 m	Exploratory SHAP-derived screening criterion	Proximity to streams below this threshold increases hotspot probability	420–580 m
Slope	<2°	Exploratory SHAP-derived screening criterion	Flat terrain (<2°) is a necessary condition for maladaptation	1.7–2.3°

Note: The flood_freq ≥ 2 threshold is the operational definition used to identify repeatedly flooded areas for all statistical analyses. The flood_freq ≥ 3 threshold, along with the DEM, distance-to-stream, and slope thresholds, are exploratory SHAP-derived screening criteria intended for risk zoning and targeted interventions. These two sets of criteria serve different purposes and should not be conflated.

## Data Availability

The original data presented in the study are openly available which can be downloaded from Supplementary Materials Files.

## Supplementary Materials

The following supporting information can be downloaded at: https://www.mdpi.com/article/10.3390/s26154853/s1. The repository includes the complete dataset (1159 spatial units with population estimates, flood records, and topographic variables) and the full Python code for data processing, trajectory classification, statistical analysis, Random Forest model training, SHAP analysis, and visualization generation.
